# Sleep-Disordered Breathing and Central Respiratory Control in Children: A Comprehensive Review

**DOI:** 10.3390/children12030279

**Published:** 2025-02-25

**Authors:** Marco Zaffanello, Angelo Pietrobelli, Luana Nosetti, Giuliana Ferrante, Erika Rigotti, Stefania Ganzarolli, Giorgio Piacentini

**Affiliations:** 1 Department of Surgery, Dentistry, Gynecology and Pediatrics, University of Verona, 37126 Verona, Italy; angelo.pietrobelli@univr.it (A.P.); giuliana.ferrante@univr.it (G.F.); erika.rigotti@aovr.veneto.it (E.R.); giorgio.piacentini@univr.it (G.P.); 2 Lombardy Regional SIDS Center, Division of Pediatrics, F. Del Ponte Hospital, University of Insubria, 21100 Varese, Italy; luana.nosetti@uninsubria.it; 3 Pediatric Division, Azienda Ospedaliera Universitaria Integrata, 37126 Verona, Italy; stefania.ganzarolli@aovr.veneto.it

**Keywords:** central respiratory, central sleep apnoea, children, infant, neurorespiratory, obstructive sleep apnoea, sleep-disordered breathing

## Abstract

**Background/Objectives**: Sleep-disordered breathing (SDB) is a primary concern in children’s health. Research suggests that repeated oxygen drops during sleep—common in SDB—may harm the brainstem’s breathing control centres. This damage likely occurs through oxidative stress, inflammation, and cell death, which weaken the brain’s ability to regulate breathing. Over time, these effects could lead to functional changes (e.g., disrupted chemical signalling) and physical damage in critical brain regions, creating a cycle of unstable breathing. However, much of this evidence comes from animal or lab studies, leaving gaps in our understanding of how these mechanisms work in humans. This review synthesises existing research on how breathing disruptions during sleep—particularly episodes of intermittent hypoxia—affect the brain’s ability to control respiration in children and adolescents. **Methods**: We analysed studies from medical databases PubMed, Scopus, and Web of Science, focusing on how SDB (obstructive or central sleep apnoea) impacts the brain’s respiratory centres in young populations. Animal studies and research involving children on mechanical ventilation were excluded to focus on natural sleep patterns. **Results**: After removing duplicates, 54 studies remained. Additionally, 43 record were excluded for various reasons. Ultimately, 11 articles were selected for the final analysis, including three that focused on genetic conditions, such as Down syndrome, Prader–Willi syndrome, and Pierre Robin sequence. The findings suggest that repeated oxygen dips during sleep may harm the brainstem’s respiratory control areas, especially during critical developmental stages. This damage could lead to long-term issues, such as unstable breathing, cardiovascular strain, or neurological problems. However, most studies only captured the immediate effects of low oxygen, leaving uncertainty about permanent harm due to a lack of long-term follow-up. **Conclusions**: Repeated oxygen deprivation during sleep appears to damage the brainstem and disrupt breathing regulation. However, small study sizes and short observation periods limit the strength of these conclusions. Future research should use advanced imaging tools to clarify long-term risks, develop effective treatments, and track children over extended periods. More significantly, longer-term studies are urgently needed to guide clinical care for vulnerable populations.

## 1. Introduction

Sleep-disordered breathing (SDB), including central sleep apnoea (CSA) and obstructive sleep apnoea (OSA), is a significant health issue in children and teens. It disrupts the balance between the nervous and respiratory systems, potentially hindering healthy development [1]. In OSA, repeated breathing pauses occur during sleep due to blocked airways or faulty brain signals controlling respiration [2].

Low oxygen levels can harm the brainstem’s respiratory centres, especially in developing children or those with genetic risks. This damage includes imbalances in brain chemicals, malfunctioning oxygen sensors, and structural changes like a buildup of star-like brain cells (astrogliosis) [3,4]. Studies also show abnormal brain development in kids with OSA, affecting areas critical for breathing [5].

The brainstem contains specialised cells that act as oxygen sensors, activating breathing when oxygen levels fall [6]. During abrupt oxygen shortages, the body responds in two distinct phases: an initial surge in breathing effort, followed by a rapid decline as the brainstem’s regulatory circuits become overwhelmed. This pattern mirrors a system pushed to its limits—briefly compensating before faltering under strain [7,8,9]. Over time, repeated oxygen deprivation can harm breathing, heart health [10], and brain function [9,11]. In adults with OSA, brain scans reveal damage to white matter—the “wiring” connecting areas like the limbic system, cerebellum, and cortex [12,13].

While SDB research has advanced, scientists still struggle to understand how the brainstem controls breathing in children. Specifically, two areas—the medulla and pons—play a critical role in maintaining breathing rhythms during sleep and adapting to chemical cues like oxygen and carbon dioxide levels in the blood. These regions act like a “breathing control centre”, fine-tuning respiration based on the body’s needs, but much about their function in kids remains unclear [14,15]. However, in kids with SDB, repeated breathing pauses may damage these areas, making them less responsive to oxygen changes or airway shifts [16,17]. For example, chronic low oxygen in OSA may blunt the brainstem’s oxygen sensors, worsening breathing instability [18].

Emerging research links SDB’s body-wide effects—like poor brain oxygenation and irregular heart function—to harmful changes in brainstem regions in paediatric patients [19,20]. These changes might explain why some children still struggle with SDB even after surgery to fix airway blockages [21,22]. This highlights the need to differentiate between physical airway issues and nervous system dysfunction when treating SDB [23,24].

### Aim of the Study

This study reviews the existing literature on the link between SDB, intermittent hypoxaemia, and the function of the neurological centres that regulate respiration during sleep in children and adolescents.

## 2. Materials and Methods

We searched three major databases—MEDLINE (PubMed), Scopus, and Web of Science—for English-language studies published from their earliest records up to 20 January 2025. Custom search terms were tailored for each database, combining the following keywords:

Inclusion terms: sleep-disordered breathing, sleep apnea, obstructive sleep apnea, upper airway obstruction paired with neurorespiratory, central respiratory, medullary respiratory, pontine respiratory, and age-related terms (children, infants, adolescents).

Exclusion terms: case reports, animal studies, mechanical ventilation techniques, and studies focused on adults or elderly populations.

## 3. Results

**Figure 1:**
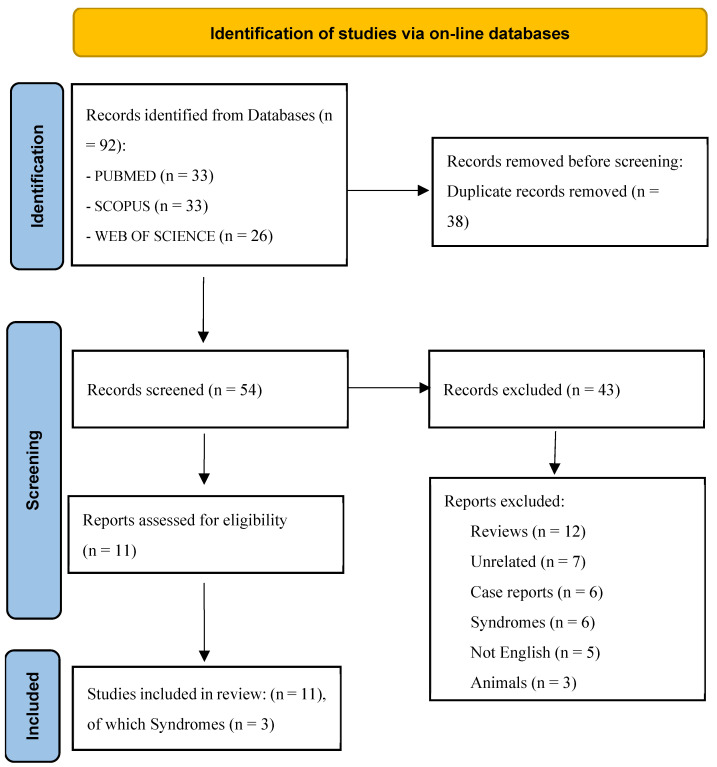
The PRISMA flow diagram visually represents the study selection process and the number of studies included at each stage. *From*: Page MJ, McKenzie JE, Bossuyt PM, Boutron I, Hoffmann TC, Mulrow CD et al. The PRISMA 2020 statement: an updated guideline for reporting systematic reviews. BMJ 2021;372:n71. doi: 10.1136/bmj.n71. For more information, visit: http://www.prisma-statement.org/ (accessed on 22 February 2025).

A total of 92 articles were retrieved from the literature search, among which 38 were identified as duplicates. As a result, 43 studies were excluded (Figure 1). Ultimately, 11 articles were selected for the final analysis, including 3 that focused on syndromic children.

Table 1 provides details on two studies investigating OSA. One notable study, a crossover trial involving 10 school-aged children [20], demonstrated that exposure to high altitude exacerbates the severity of SDB. Specifically, the study reported an increased AHI, worsening oxygenation (SpO_2_), increased sleep fragmentation, and heightened cardiovascular stress, as indicated by elevated heart rate [20]. The findings indicate that the low-oxygen conditions of high-altitude environments (hypobaric hypoxia) might interfere with the brain’s ability to regulate breathing—specifically disrupting communication between the medulla and pons, key brainstem regions that act as the body’s “breathing command centre.” Surprisingly, despite the strain on the body, the study found no apparent harm to children’s brain function or heart health [20]. The researchers speculated that this could be because the children were only exposed briefly or because their bodies adapted temporarily to protect them.

While the study confirmed physical changes like reduced oxygen levels and fragmented sleep, it did not detect clear signs of long-term neurological or cardiovascular damage. This gap between observed stress and visible harm raises questions about whether children’s resilience or the study’s limitations (like short-term observation) masked subtle effects. This discrepancy suggests that children may possess transient adaptive mechanisms that mask potential subclinical neuronal damage [20]. Furthermore, the value of monitoring Tissue Oxygenation Index (TOI) and pulse transit time (PTT) in children [19] supports using portable, multimodal assessment tools to evaluate SDB severity and central nervous system (CNS) involvement in real-world settings.

Another study examined a cohort of 60 children aged 3 to 12, revealing that central respiratory events during non-REM (NREM) sleep led to reductions in cerebral oxygenation like those observed in obstructive events [19]. These episodes coincided with changes in peripheral blood pressure and heart rate, suggesting that central apnoea might lead to brief periods of cerebral hypoxia [19]. This observation supports previous findings showing that intermittent hypoxia, common in SDB, can harm neurological development and cardiovascular health, even without obvious immediate symptoms. PTT, a non-invasive measure of autonomic activity, significantly drops during breathing disruptions, reflecting increased sympathetic nervous system activity [19]. Similarly, the TOI falls during apnoea events, showing reduced oxygen supply to tissues and underscoring how respiratory regulation affects overall blood flow [19]. These physiological markers provide insight into how central respiratory control dysfunction can trigger a cascade of autonomic and cardiovascular responses mediated by brainstem nuclei and higher cortical networks. Finally, the finding that central and obstructive respiratory events similarly reduce cerebral oxygenation [19] underscores the need for a shift in perspective regarding SDB management.

Table 2 provides an overview of studies examining the effects of predominantly central SDB on sleep physiology in children, comprising three studies focusing on infants and three on older children, all within specific clinical contexts.

A histopathological investigation of the medulla oblongata in newborns showed notable changes in dendritic spine density within respiratory control centres before and after birth [25]. These results emphasise the importance of neuronal maturation in establishing robust respiratory regulation, highlighting how early susceptibility to SDB may stem from the developmental progression of these critical neural networks [25].

An experimental approach was proposed to counteract central respiratory insufficiency in neonates, using a combination of sensory stimuli, including light exposure and the controlled modulation of O_2_ and CO_2_ levels, to stabilise breathing during sleep [26]. While promising, this technique requires further clinical studies to assess its efficacy in preventing hypoxaemia in high-risk newborns [26].

A higher incidence of CA was observed in neonates classified as small for gestational age (SGA), attributing this phenomenon to neurological alterations associated with intrauterine growth restriction (IUGR) [27]. These findings highlight how prenatal factors influence respiratory stability after birth and the likelihood of developing SDB [27]. Progress in diagnostic tools has been driven by Fukumizu (2004) and Foo et al. (2005, 2008), who confirmed that PTT reliably detects central breathing disruptions in children non-invasively [28,29,30]. Their work marks significant progress in bridging research and clinical practice, offering practical methods to monitor how breathing and brain activity interact during sleep without disturbing natural sleep patterns.

Table 3 shows the effects of SDB, particularly sleep apnoea, on central respiratory control and its interactions with the nervous and cardiovascular systems, with special attention to the paediatric implications of the studies summarised in Table 2.

The crucial role of dendritic spine development in the respiratory centres of the medulla oblongata has been highlighted, linking neuronal maturation to respiratory control in neonates. This study provides valuable insights into the mechanisms underlying primary apnoea in preterm infants and sleep apnoea associated with sudden infant death syndrome (SIDS), reinforcing the importance of neurodevelopmental monitoring in high-risk neonates [25].

An innovative method to prevent low oxygen levels in newborns was proposed, involving sensory interventions like light exposure and adjustments to breathing gases, paired with the precise tracking of oxygen and carbon dioxide levels. Though promising, more clinical trials are needed to verify its usefulness in newborn care [26].

Infants born SGA showed more frequent breathing pauses, likely due to immature respiratory centres associated with growth restriction during pregnancy. This research stresses the importance of specialised respiratory monitoring for these high-risk infants [27].

The connection between central breathing irregularities (like pauses and sighs) and the brain’s control of respiration has been explored, revealing that breathing regulation shifts with age and sleep phases. These insights reinforce the idea that respiratory control evolves dynamically as the nervous system matures [28].

Finally, studies by Foo et al. validated the use of plethysmography and PTT as non-invasive tools for studying CSA. Their work has helped clarify the interactions between respiratory variability, oxygenation, and arterial stiffness, contributing to improved research methodologies for SDB in paediatric populations [29,30].

The evidence in Table 4 shows how SDB affects the brain’s control of breathing in children with genetic syndromes, exploring how genes, body structures, and bodily functions contribute to these challenges.

In children with Prader–Willi syndrome, sleep studies by Schlüter et al. (1997) found that the main issue lies in how the brain regulates breathing, likely due to faulty brain signals and physical airway blockages [31]. Similarly, research comparing children with Down syndrome to others revealed that CSA is more common in this group, possibly tied to impaired brainstem function [32]. These findings illustrate how genetic risks and physical problems interact to worsen SDB.

A study on children with Pierre Robin sequence [33] observed uncoordinated breathing muscle activity during sleep, even when the brainstem appeared normal. This suggests that respiratory issues in some syndromes may stem from glitches in how specific brain networks operate rather than visible structural damage [33].

These patterns mirror what doctors see in children with brainstem injuries—whether present at birth or occurring later—where breathing pauses and shallow breathing are common. Such insights stress the importance of specialised care and close breathing monitoring in these vulnerable groups.

## 4. Discussion

Existing evidence suggests that even brief bouts of hypoxia—such as those encountered at high altitudes—can significantly worsen SDB indices (for instance, the AHI), increase sleep fragmentation, and place a heavier strain on the cardiovascular system in paediatric patients [20]. However, in adults, OSA becomes CSA as chemoreceptor sensitivity increases, and central respiratory motor output instability occurs during a sojourn at high altitudes [34]. These findings point to the possibility that hypobaric hypoxia may disrupt respiratory control mechanisms at the level of the brainstem.

Studies in children using advanced measurement techniques (including TOI and PTT) also indicate that both CSA and OSA can lower cerebral oxygenation and activate the sympathetic nervous system, demonstrating that central events—often considered less severe—can trigger notable autonomic and cardiovascular responses [19,29,30].

Furthermore, breathing regulation in infancy and childhood involves substantial maturational and structural components [26,27,28,29,30]. Histopathological data highlight the critical role of dendritic development in the bulbar centres. At the same time, prenatal factors (such as IUGR, leading to SGA status) appear to increase the likelihood of respiratory irregularities in paediatric age, including premature babies [25,27]. Finally, children with specific genetic syndromes—such as Prader–Willi [31], Down [35], or Pierre Robin sequence [33]—have been shown to experience dysfunctional central respiratory control stemming from both structural and functional abnormalities.

However, the heterogeneity of the included studies, such as small sample sizes [20,29,31,32] and varying methodologies (PSG, transcutaneous pO_2_ monitoring, plethysmography, and PTT analysis), limits the generalizability of the results. Additionally, the scarcity of neuroimaging data in most studies precludes a direct correlation between physiological markers and brainstem alterations.

The exacerbation of SDB at high altitudes [20] underscores the sensitivity of paediatric respiratory centres to hypobaric hypoxia. Observed increases in the AHI, sleep fragmentation, and cardiovascular stress in this context suggest that hypoxia compromises the function of medullary chemoreceptors in experimental data [36,37] and the integration of autonomic feedback at the pons in models [38] and mammals [39], both crucial for maintaining rhythmic breathing [40]. The absence of overt neurocognitive deficits in this cohort could reflect transient adaptive plasticity, such as increased serotonergic signalling in the brainstem in the mouse model [41] and temporarily stabilising respiratory drive in models [18]. However, prolonged or recurrent exposure to hypoxia could overwhelm compensatory capabilities in adults [42] and using animal models [43], in line with evidence suggesting that intermittent hypoxia induces oxidative stress in brainstem neurons [44], impairing synaptic plasticity over time [45].

While OSA is often seen as a problem of blocked airways, the issue goes beyond anatomy. The nervous system’s ability to maintain steady airflow is equally essential [46]. For instance, the brainstem’s emergency wake-up reaction—triggered by low oxygen—interrupts healthy sleep patterns [47], creating a harmful cycle of oxygen drops and abrupt awakenings [2,39]. Over time, these repeated disruptions may gradually alter brain structure, shrinking grey matter in critical regions like the prefrontal cortex, hippocampus, and cerebellum, affecting thinking, behaviour, and learning [13,48].

During non-REM sleep, CSA reduces brain oxygen levels like OSA [49], questioning the traditional focus on fixing airway mechanics alone in children [19]. This hints that flaws in brain networks governing breathing rhythms and arousal—specifically in the pons and medulla—might explain OSA and CSA [50,51]. Post-mortem studies support this idea, showing that brainstem damage directly disrupts breathing control [52], while brain scans reveal weaker connections between the pons and prefrontal cortex in people with CSA [53].

The dynamic development of medullary respiratory nuclei indicates a critical phase in childhood where the formation of dendritic spines is essential for establishing stable respiratory control [25]. Disruptions in this process, such as those caused by IUGR [27], can predispose infants to CSA, highlighting the long-term impact of prenatal insults on respiratory stability [54]. Similarly, the efficacy of sensory stimulation in reducing hypoxemia [26] suggests that immature brainstem circuits in infants can be externally modulated [55,56]. However, the clinical translation of these techniques requires further validation.

In children with Prader–Willi syndrome, two mechanisms underlie SDB [31]: mechanical obstruction of the airways and reduced CO_2_ sensitivity in medullary chemoreceptors. This duality mirrors observations in the Pierre Robin sequence [33], where the functional disorganisation of pontomedullary networks, rather than overt structural anomalies, has been identified as the cause of respiratory dysfunction. These studies demonstrate that SDB arises from a complex interaction between brainstem dysfunctions and anatomical vulnerabilities in genetic syndromes [57]. In adult patients (aged 47.8 ± 12.3 years) with genetic abnormalities, the instabilities in the ventilatory control system can generate the temporary arrest of the respiratory drive (CSA), which is also a consequence of the obstruction of the airway [17]

In paediatric age, it has been observed that PTT and TOI [28,29,30] reflect the integration between autonomic responses and CNS involvement in SDB. A reduction in PTT during apnoea indicates sympathetic activation [58], while changes in TOI highlight alterations in oxygen distribution, both processes mediated by brainstem nuclei. The combined use of these tools and home PSG in children [20] allows for assessing SDB severity and central involvement in real-world contexts, bridging the gap between laboratory research and clinical practice.

Current evidence supports the hypothesis that intermittent hypoxia in children with OSA may contribute to damage to the brainstem respiratory centre through oxidative stress [59], inflammation [60,61], and apoptosis, leading to compromised ventilatory control. Chronic intermittent hypoxia promotes a pro-oxidative state that destabilises rhythmogenesis in the preBötzinger complex, leading to sporadic failures in transmission to the XII nerve. These effects could perpetuate apnoea and respiratory instability [62]. However, causality is often inferred from animal models [63,64,65], where the roles of oxidative stress and apoptosis-related neural injury are confirmed [66]. Therefore, OSA worsens neurological outcomes in mice and increases neuronal death by enhancing neural apoptosis and neuroinflammation [67].

This comprehensive body of evidence—spanning pathophysiology, neurophysiological parameters, and clinical observations—clearly shows how intermittent hypoxia and SDB can disrupt the normal functioning of the brainstem’s respiratory centres (medulla oblongata and pons). Children can be especially vulnerable, with these alterations often manifesting during sleep.

Repeated episodes of low oxygen (or intermittent hypoxia) can directly damage the brainstem regions responsible for controlling breathing and lead to broader health issues that weaken the body’s ability to regulate respiration. Over time, these disturbances may disrupt sleep and waking hours, making it harder to maintain steady breathing patterns.

This study faces some important limitations, starting with the limited number of available studies and the differences in research protocols, such as variations in diagnostic criteria, sample sizes, and population characteristics. These factors make it difficult to draw definitive conclusions. Another challenge is that most existing research is based on short-term observations and lacks advanced neuroimaging techniques. This would help provide a clearer understanding of how SDB affects central respiratory control. Many studies also rely on indirect physiological markers like TOI or PTT. While these tools are valuable and non-invasive, they may not be enough to capture the complexity of brainstem function in children fully.

A standardised approach is essential to ensure that studies produce reliable and comparable results, ultimately driving progress in this field. Another key step is the use of advanced neuroimaging techniques, such as MRI and functional MRI, which can provide deeper insights into the structural and functional changes affecting the brainstem and related areas. Expanding research through large-scale, long-term, multicentre studies would help paint a more comprehensive picture of both the immediate and long-term neurological effects of sleep-disordered breathing (SDB).

Collecting detailed data on factors that influence respiratory control—such as coexisting conditions or genetic syndromes—would also be crucial in helping researchers gain a more accurate understanding of the true impact of these disorders.

When SDB is caused by anatomical obstructions, such as adenotonsillar hypertrophy or craniofacial anomalies, targeted interventions can significantly improve nighttime breathing. However, these treatments may not fully address underlying dysfunctions in central respiratory control. In cases where neurological impairment plays a dominant role, as seen in genetic syndromes or brainstem abnormalities, focusing solely on airway management may not be sufficient. Moreover, it is important to recognise that repeated episodes of intermittent hypoxia caused by SDB could further worsen pre-existing dysfunctions in central respiratory control, making it even harder for the body to regulate breathing effectively.

While numerous studies focus on various interventions in treating OSA (anti-inflammatory drugs, surgery for adenotonsillar hypertrophy, or CPAP), little evidence supports therapies that directly modulate central respiratory drive in children. Some isolated neonate reports have evaluated sensory stimulation approaches—combining specific light exposure with controlled O_2_/CO_2_ adjustments [68,69]. However, these methods remain experimental [26]. Likewise, some paediatric studies have investigated the use of respiratory stimulants, such as caffeine, for apnoea of prematurity [70]. However, these approaches have not been considered for older children with OSA or central apnoea.

In children with genetic conditions involving abnormal brainstem function (e.g., Prader–Willi syndrome or Down syndrome), addressing structural airway issues may partially alleviate SDB [71,72] but often does not fully correct the underlying central component [31,32]. Consequently, while non-invasive ventilation (e.g., BiPAP) may compensate for the inadequate respiratory central drive [73], evidence supporting solid neurologic therapy (e.g., pharmaceuticals or neuromodulation devices) in the paediatric population remains insufficient. Large-scale, long-term studies utilising advanced neuroimaging are needed to determine whether future therapeutic approaches for central respiratory control (e.g., brainstem-targeted drugs, sensory stimulation, or neural pacing devices) could be effective and safe for children [20,26].

Given the combined anatomical and neurological factors involved in SDB, a multidisciplinary approach is essential, involving specialists in paediatrics, otolaryngology, neurology, and sleep medicine. Tailoring treatment to the patient’s underlying cause will likely yield better clinical outcomes than a standardised approach.

Identifying and treating the root causes of intermittent hypoxia is essential to safeguard the development and function of these critical breathing control areas in children.

## 5. Conclusions

Research shows that even short periods of low oxygen—like those occurring at high altitudes—can worsen markers of SDB, disrupt sleep quality, and put extra stress on the heart and blood vessels. These effects may weaken the brainstem’s ability to regulate breathing. Both types of breathing pauses (central and obstructive) reduce oxygen flow to the brain and spark overactive “fight-or-flight” nervous system responses, with children being particularly vulnerable. Given the limitations of existing studies in children, future research—using tools like advanced brain imaging—is crucial to fully grasp these issues’ lasting impacts and create better prevention and treatment plans.

## Figures and Tables

**Table 1:** Summary of included studies and key findings.

Author(s) (Year of Publication)	Country	Design	Subjects	Methods	Results	Conclusions	Relationship Between SDB/OSA and Neurology
Hughes BH (2017) [20]	U.S.A. (Colorado)	Crossover study	10 children (4.9 to 12.7 years; 60% males); high altitude; SDB	Home PSG (high altitude) vs. laboratory PSG (low altitude) comparison	Elevated AHI, worse oxygenation, increased heart rate, and sleep fragmentation at high altitude (AHI 10.95/h, ODI 19.5/h) vs. low altitude (AHI 2.4/h, ODI 3.6/h)	High altitude worsens the symptoms of SDB; home tests more indicative of severity	Neurophysiological impact; altitude; severity of SDB; major neurological impairment
Tamanyan K (2019) [19]	Australia	Observational study	60 children (3–12 years) with suspected SDB	PSG, detailed analysis of cerebral and peripheral oxygenation, blood pressure, heart rate changes	Greater variation in cerebral oxygenation during central respiratory events compared to obstructive ones, especially in NREM sleep	Central respiratory events affect cerebral oxygenation like obstructive events	Significant impact of CA on neurocognitive and cardiovascular health like obstructive events
Authors and Date		Type of Apnoea	Apnoea Assessment Methods	Impact on Respiratory Centres and CNS	Evaluation of Respiratory Centres and CNS	Clinical Implications	Conclusions
Hughes BH (2017) [20]	U.S.A. (Colorado)	Obstructive and central apnoea	AHI, SpO_2_, CAI, CHI	Altitude exacerbates the severity of SRBDs. It influences central breathing control and cardiovascular stress.	AHI, SpO_2_, heart monitoring	High altitude increases respiratory interruptions and lowers SpO_2_, impacting CNS and cardiovascular health.	Environmental factors such as altitude critically influence the severity of SRBDs and their physiological impact on the respiratory and cardiovascular systems. Need for specific strategies for altitude.
Tamanyan (2019) [19]	Australia	Central and obstructive sleep apnoea	TOI, PTT, HR monitoring	Changes in TOI, PTT, and HR during respiratory events reflect the role of the CNS and the autonomic nervous system in regulating cardiovascular and respiratory responses.	TOI, PTT, HR monitoring during sleep, differentiation between REM and NREM sleep	Significant physiological changes do not result in clinically meaningful findings, underscoring the complexity of diagnosis and treatment based on physiological responses alone.	Need for more refined methodologies to differentiate the impacts of various types of respiratory events on physiological parameters and implications for respiratory control and CNS in children.

Legend: AHI, Apnoea–Hypopnoea Index; CA, central apnoea; CAI, Central Apnoea Index; CHI, Central Hypopnoea Index; CNS, central nervous system; HR, heart rate; NREM, non-rapid eye movement; ODI, Oxygen Desaturation Index; OSA, obstructive sleep apnoea; SpO_2_, oxygen saturation; PSG, polysomnography; PTT, pulse transit time; REM, rapid eye movement; SDB, sleep-disordered breathing; SRBDs, sleep-related breathing disorders; TOI, Tissue Oxygenation Index.

**Table 2:** Summary of studies on central sleep-disordered breathing and sleep physiology in children.

Author(s) (Year of Publication)	Country	Design	Subjects	Methods	Results	Conclusions	Relationship Between SDB/OSA and Neurology
INFANTS							
Takashima S. and Becker L.E. (1986) [25]	Canada	Observational study	26 individuals, foetuses (gestational age 18 to 42 weeks) and newborns (from 0 to 11 months)	Neurons in the medulla oblongata Golgi impregnation methods	Increase in pre-birth dendritic spines, decrease post-birth	Maturation of respiratory neurons occurs in prenatal and postnatal stages	Development of central respiratory control influenced by dendritic changes, implications for SIDS and apnoea of prematurity
Schlaefke M.E. et al. (1987) [26]	Germany	Experimental study	30 patients aged 6 to 78 months	Paired stimuli (light and O_2_/CO_2_), transcutaneous pO_2_ monitoring	Effective prevention of sleep hypoxemia in unventilated infants	Potential preventive/therapeutic approach for central respiratory failure and sleep apnoea	Direct intervention on central respiratory control mechanisms can modify neurological responses
Curzi-Dascalova L. et al. (1996) [27]	France	Observational study	57 infants (31 AGA and 26 SGA)	Postnatal polygraphic records	SGA infants show more central breathing pauses than AGA	Impaired respiratory control in SGA infants; possible brainstem changes	Neurological changes from IUGR affect central respiratory control
CHILDREN							
Fukumizu and Kohyama (2004) [28]	Japan	Clinical trial	19 healthy children (3 months–7 years, mean 28 months)	PSG, respiratory plethysmography	Correlation between sighs, body movements, and central breathing pauses	Age- and status-related changes in breathing pauses	CSA related to central sleep disorders
Foo JY (2005) et al. [29]	Australia	Experimental study	5 infants (mean age 7.8 months)	PSG, PTT analysis	PTT sensitive in detecting central respiratory events	PTT as a non-invasive method of monitoring CA	Detection of central respiratory events in newborns
Foo JY et al. (2008) [30]	Singapore	Observational study	28 children (age 6.2 ± 3.6 years)	PTT analysis during CSA and tidal breathing	Differences in PTT oscillations between CSA and normal breathing; PTT increase during clustered CSA	Predictive models for characteristics of respiratory events	Changes in vascular dynamics during sleep events reflect neurological and respiratory control alterations

Legend: AGA, appropriate for gestational age; AHI, Apnoea–Hypopnoea Index; CA, central apnoea; CSA, central sleep apnoea; IUGR, intrauterine growth restriction, OAHS, obstructive sleep apnoea–hypopnoea syndrome; PSG, polysomnography; PTT, pulse transit time; RP, respiratory polygraphy; SDB, sleep-disordered breathing; SGA, small for gestational age; SIDS, sudden infant death syndrome.

**Table 3:** Literature review on sleep-disordered breathing and its impact on central respiratory control.

Author(s) (Year of Publication)	Type of Apnoea	Apnoea Assessment Methods	Impact on Respiratory Centres and CNS	Evaluation of Respiratory Centres and CNS	Clinical Implications	Conclusions
INFANTS						
Takashima and Becker (1986) [25]	Primary apnoea in prematurity, SIDS	Evaluation of neuronal development	Development of dendritic spines related to maturation of respiratory control	Examination of the density and maturation of dendritic spines	Potential use of neuronal development models as biomarkers or therapeutic targets	Neuronal development in the medulla oblongata crucial for respiratory control; important for research and preventive interventions
Schlaefke ME (1987) [26]	CSA, Ondine syndrome	Therapy with paired stimuli, transcutaneous pO_2_ monitoring, and end-tidal pCO_2_	Therapy stimulates respiratory responses, preventing hypoxemia	Monitoring of pO_2_ and pCO_2_ levels, PSG	Therapeutic and preventive potential for SIDS and CSA	Efficacy of targeted therapeutic stimulation in improving conditions of central respiratory insufficiency
Curzi-Dascalova L. (1996) [27]	Apnoea in SGA infants	Not specified	Increased breathing pauses and apnoea index indicate impairment of respiratory centres	Comparison of periodic breathing patterns and developmental parameters	Importance of monitoring and supporting respiratory functions in SGA infants	Complex interplay between delayed growth, respiratory control, and CNS development
CHILDREN						
Fukumizu and Kohyama (2004) [28]	Sleep apnoea, OSA	Observation of central pauses, sighs, and gross movements during sleep	Central respiratory events related to neurological controls of breathing, influenced by age and sleep stage	Analysis of patterns of respiratory events and their correlation with neurological development	Need to understand age- and sleep stage-related changes to manage respiratory disorders	Importance of targeted therapeutic strategies based on sleep development and behaviour
Foo JY et al. [29,30]	CSA	Respiratory inductance plethysmography, PTT analysis	Decreased variability and command during episodes of apnoea, indicating less active neurological control	PTT analysis during normal breathing cycles and CSA	Reduced breathing effort and alterations in oxygen saturation during apnoea can compromise tissue oxygenation	Importance of targeted treatment strategies to improve respiratory regulation and patient outcomes

Legend: CNS, central nervous system; CSA, central sleep apnoea; OSA, obstructive sleep apnoea; PTT, pulse transit time; PSG, polysomnography; SGA, small for gestational age; SIDS, sudden infant death syndrome.

**Table 4:** Impact of sleep-disordered breathing on central respiratory control in children with genetic syndromes.

Author(s) (Year of Publication)	Aims	Subjects	Methods	Results and Conclusions	Relationship Between SDB/OSA and Neurology
Schlüter B et al. (1997) [31]	Respiratory control in Prader–Willi syndrome	8 patients (6 weeks–12.5 years)	Comparative study with PSG	Primary central respiratory control disorder in Prader–Willi syndrome, aggravated by obesity	It shows a direct relationship between central respiratory dysfunction and the presence of SDB in Prader–Willi syndrome
Ferri R et al. (1997) [32]	Breathing patterns during sleep in Down syndrome	10 subjects (8.6–32.2 years)	Comparative study	Higher prevalence of CSA in Down syndrome, suggesting brainstem dysfunction	Links CSA to central respiratory control dysfunctions in brainstem Down syndrome
Renault F et al. (2000) [33]	Neurophysiological investigations of the brainstem in the Pierre Robin sequence	25 newborns (age not available)	Observational study with PSG and EMG	Functional disturbances in motor organisation affect breathing but absence of structural damage to the brainstem	Despite the absence of structural damage, functional motor control disorders contribute to SDB

Legend: CSA, central sleep apnoea; EMG, electromyography; PSG, polysomnography; SDB, sleep-disordered breathing.

## Data Availability

Data are derived from public domain resources.
